# Association of Online Health Information–Seeking Behavior and Self-Care Activities Among Type 2 Diabetic Patients in Saudi Arabia

**DOI:** 10.2196/jmir.4312

**Published:** 2015-08-12

**Authors:** Amr Jamal, Samina A Khan, Ahmed AlHumud, Abdulaziz Al-Duhyyim, Mohammed Alrashed, Faisal Bin Shabr, Alwalid Alteraif, Abdullah Almuziri, Mowafa Househ, Riaz Qureshi

**Affiliations:** ^1^ College of Medicine Medical Informatics and E-Learning Unit, Medical Education Department King Saud University Riyadh Saudi Arabia; ^2^ College of Medicine Family and Community Medicine Department King Saud University Riyadh Saudi Arabia; ^3^ KSU Chair for Medical Education Research and Development King Saud University Riyadh Saudi Arabia; ^4^ College of Medicine King Saud University Riyadh Saudi Arabia; ^5^ College of Public Health and Health Informatics King Saud Bin Abdulaziz University for Health Sciences, Ministry of National Guard Health Affairs (MNGHA) Riyadh Saudi Arabia

**Keywords:** Internet, diabetes mellitus, type 2, self-care, consumer health information, telemedicine, medical informatics, health education, Google, eHealth, e-patients, health behavior, Middle East, Saudi Arabia

## Abstract

**Background:**

Health information obtained from the Internet has an impact on patient health care outcomes. There is a growing concern over the quality of online health information sources used by diabetic patients because little is known about their health information–seeking behavior and the impact this behavior has on their diabetes-related self-care, in particular in the Middle East setting.

**Objective:**

The aim of this study was to determine the online health-related information–seeking behavior among adult type 2 diabetic patients in the Middle East and the impact of their online health-related information–seeking behavior on their self-care activities.

**Methods:**

A cross-sectional survey was conducted on 344 patients with type 2 diabetes attending inpatient and outpatient primary health care clinics at 2 teaching hospitals in Riyadh, Saudi Arabia. The main outcome measures included the ability of patients to access the Internet, their ability to use the Internet to search for health-related information, and their responses to Internet searches in relation to their self-care activities. Further analysis of differences based on age, gender, sociodemographic, and diabetes-related self-care activities among online health-related information seekers and nononline health-related information seekers was conducted.

**Results:**

Among the 344 patients, 74.1% (255/344) were male with a mean age of 53.5 (SD 13.8) years. Only 39.0% (134/344) were Internet users; 71.6% (96/134) of them used the Internet for seeking health-related information. Most participants reported that their primary source of health-related information was their physician (216/344, 62.8%) followed by television (155/344, 45.1%), family (113/344, 32.8%), newspapers (100/344, 29.1%), and the Internet (96/344, 27.9%). Primary topics participants searched for were therapeutic diet for diabetes (55/96, 57%) and symptoms of diabetes (52/96, 54%) followed by diabetes treatment (50/96, 52%). Long history of diabetes, familial history of the disease, unemployment, and not seeking diabetes education were the most common barriers for online health-related information–seeking behavior. Younger age, female, marital status, higher education, higher income, and longer duration of Internet usage were associated with more online health-related information–seeking behaviors. Most (89/96, 93%) online health-related information seekers reported positive change in their behaviors after seeking online health information. Overall odds ratio (OR 1.56, 95% CI 0.63-3.28) for all self-care responses demonstrated that there was no statistically significant difference between those seeking health-related information online and non–health-related information seekers. However, health-related information seekers were better in testing their blood glucose regularly, taking proper action for hyperglycemia, and adopting nonpharmacological management.

**Conclusions:**

Physicians and television are still the primary sources of health-related information for adult diabetic patients in Saudi Arabia whether they seek health-related information online or not. This study demonstrates that participants seeking online health-related information are more conscious about their diabetes self-care compared to non–health-related information seekers in some aspects more than the others.

##  Introduction

The expansion of the Internet has enabled people all over the world to gain access to a substantial amount of information on a variety of topics related to health sciences, human sciences, literature, and history [1,2]. Today, in the era of information technology, diabetic patients have become more dependent on online sources to access health information ubiquitously, especially with the propagation of smartphones, tablets, and laptops. Health-related information on the Internet for diabetes encompasses thousands of websites, chat rooms, and support groups that can be accessed by health consumers [2,3]. The medical community has studied the positive effects that online health-related information can have on patients, especially diabetic patients [4-7]. Previously published studies related to health information-seeking behaviors of diabetic patients have also addressed the potential benefits of online health-related information accessibility for diabetic patients as they search for information and advice about symptoms, disorders, and their appropriate treatments for diabetes [4-7]. An increasing number of patients are searching online for health information related to diabetes. Many of these patients have low health literacy levels and may retrieve inaccurate, incomplete, or out-of-date health information. [2,8,9]. Despite potential risks associated with online health-related information, millions of people use the Internet to search for diabetes-related health information. A recent study on the health-related information-seeking behaviors of a diabetes online community found that users engaged in peer support, advocacy, self-expression, humor, sharing, and seeking diabetes information [10]. The study also reports on the potential risks for diabetic patients searching for health-related information, which includes misinformation and privacy risks. The study recommends that although the Internet provides opportunities for communication between diabetic patients and health care providers, more research is needed to investigate the impact of health-related information on diabetes self-care [10].

In Saudi Arabia, Internet usage is rapidly growing and already slightly more than half of the population is using Internet [11,12]. The practice of using the Internet to seek health-related information is also common among patients in Saudi Arabia [2]. One of the most common and disabling diseases that patients need health-related information on is type 2 diabetes mellitus. The prevalence of type 2 diabetes mellitus in Saudi Arabia is worrying because already 20% of the adult population has this disease and it is expected to exceed to 25% by 2035 [13,14]. A national multistage survey study conducted in 2013 on 10,735 Saudi participants aged 15 years or older reported a high prevalence of diabetes (13.4%). A large proportion (43.6%) of diabetic individuals were undiagnosed before and only 29.1% of those receiving treatment had controlled diabetes. In addition, 15.2% were borderline diabetic. These numbers are alarming because they indicate a total of 1,745,532 diabetic and 979,953 borderline diabetic Saudis [15]. According to the International Diabetes Federation (IDF), Saudi Arabia has the fastest rate of growth of diabetes among the Middle East and North Africa (MENA) countries and the seventh highest in the world [13]. However, despite the high penetration of the Internet in Saudi society, there is a scarcity of existing research on the effect of diabetes health-related information-seeking behavior and its impact on self-care. The purpose of this study is to (1) determine online health-related information-seeking behavior among Saudi adult patients diagnosed with type 2 diabetes and (2) evaluate the impact of online health-related information-seeking behavior among diabetic patients on their self-care.

##  Methods

### Study Design

The data for the current study were derived from a hospital-based cross-sectional survey conducted on a convenience sample of adult Saudi male and female patients diagnosed with type 2 diabetes in an outpatient and inpatient setting.

###  Setting

The study was conducted at King Saud University Medical City, Riyadh, Saudi Arabia, from February 28 to the end of March 2013. King Saud University Medical City consists of 2 teaching hospitals, which are tertiary referral hospitals with major primary health care outpatient/inpatient departments and serves patients of all sociodemographic levels in Riyadh and other parts of country. Thus, a person in Saudi Arabia can use their services freely without restriction to a specific catchment area.

### Participants

The target population was patients aged 16 years or older diagnosed with type 2 diabetes according to the American Diabetes Association’s *Standards of Medical Care in Diabetes* guidelines [16]. All medical staff caring for diabetic patients (eg, physicians, nurses, technicians) was excluded from the study. Additionally, those who were not fluent in Arabic or English language were also excluded.

### Sample Size

The diabetic patients were enrolled from outpatient and inpatient areas of the previously mentioned hospitals. The single proportion formula was used to calculate the sample size with 95% confidence level and 5% confidence interval [2]. The total sample size targeted in data collection of the present study was 344 diabetic patients.

### Data Collection

The study instrument was a structured questionnaire developed in both English and the Arabic language and was adapted from previous work. The questionnaire included the following sections: (1) demographic information, (2) general Internet usage, (3) online health-related information–seeking behavior, and (4) questions related to self-care. The survey questionnaire was pilot-tested on 20 hospital volunteers at King Khalid University Hospital, Riyadh, to determine participants’ level of comprehension. The results of the pilot study have not been included in this paper. Our trained team of researchers completed the survey by interviewing the participants individually in their preferred language of Arabic or English. The survey was conducted between February 28 and March 31, 2013. The questionnaire was validated through the pilot study feedback. A Cronbach alpha of greater than .6 was also determined for the instrument reliability.

### Data Analysis

The study data were collected and entered into a computer using standardized entry codes. For all tests, statistical significance was set at *P*<.05. Descriptive statistics were used to present means, standard deviations, and percentages. In addition, Student *t* test, *z* proportional test, and chi-square tests were employed to compare group variables between sexes, age groups, and other demographic variables. The relationships between demographic and Internet search for health-related information / self-care were assessed using binary unconditional multiple logistic regression analysis. The questionnaires were converted into binary data to run binary logistic regression. Adjusted odds ratios and the corresponding 95% confidence intervals (CIs) were calculated for each independent variable. Modeling was performed with the goal of selecting the most parsimonious and reasonable explanatory model that explained the relationship between independent and dependent variables. For bivariate analyses, all available data points were utilized. However, for multivariable analyses (logistic regression), a dataset was constructed that only had complete values for all relevant variables across the observations, thereby discarding the observations that had missing values for any of the variables involved in the regression analysis. This strategy was adopted to maintain comparability between models so that they could be developed from the same denominator. Factor subgroups were recombined for use in logistic regression analysis to prevent quasi-separation of cells resulting from compact cell sizes, which allowed the models to converge and yet provided for meaningful analyses. All analyses were conducted in SPSS version 21 (SPSS Inc, Chicago, IL, USA).

### Ethics Statement

All participants were informed about the aim of the study and their verbal consent for participation was recorded. The study was approved by the Institutional Review Board at the College Of Medicine, King Saud University, Riyadh, Saudi Arabia, and was conducted in accordance with the declaration of Helsinki for Human Studies [3].

## Results

### Response Rate and Demographic Information

Of the 394 patients we approached to participate in the study, 344 (87.3%) completed the survey, whereas 37 of 394 (9.4%) participants decided not to take part in survey due to a concurrent scheduled physician appointment and 13 of 394 (3.3%) withdrew from the study due to lack of time to complete the survey.

Of the 344 diabetes patients who were interviewed, 255 (74.1%) were males. The overall mean age of participants was 53.5 (SD 13.8) years (males: mean 54.2, SD 14.1, range 16-84 years; females: mean 51.5, SD 12.9, range 19-80 years). Further demographic features are presented in Table 1.

**Table 1:** Sociodemographic details of samples based on age group and gender.

Sociodemographic characteristics		Total, n (%) N=344	Age band, n (%)			
			≥45 years n=255		<45 years n=89	
			Male n=192	Female n=63	Male n=63	Female n=26
**Patient place**						
	Outpatient	302 (87.2)	169 (88.1)	55 (87)	54 (86)	24 (92)
	Inpatient	42 (12.2)	23 (11.9)	8 (13)	9 (14)	2 (8)
**Diabetes duration**						
	1-5 years	85 (24.7)	35 (18.2)	17 (27)	22 (35)	11 (42)
	6-10 years	71 (20.6)	39 (20.3)	15 (24)	19 (30)	10 (39)
	11-15 years	105 (30.5)	41 (21.4)	15 (24)	13 (21)	2 (8)
	≥16 years	83 (24.1)	77 (40.1)	16 (25)	9 (14)	3 (12)
**Are any of your first-degree relatives diabetic?**						
	Yes	267 (77.6)	143 (74.5)	52 (83)	50 (79)	22 (85)
	No	77 (22.4)	49 (25.5)	11 (18)	13 (21)	4 (15)
**Marital status**						
	Single	26 (7.6)	2 (1.0)	1 (2)	17 (27)	6 (23)
	Married	291 (84.6)	187 (97.4)	42 (67)	46 (73)	16 (62)
	Divorced	1 (0.3)	1 (0.6)	2 (3)	0 (0)	3 (12)
	Widowed	0	2 (1.0)	18 (29)	0 (0)	1 (4)
**Educational level**						
	Intermediate school or lower	126 (36.6)	67 (34.9)	50 (79)	4 (6)	5 (19)
	High school	112 (32.5)	67 (34.9)	11 (17)	23 (37)	11 (42)
	University	94 (27.3)	49 (25.5)	2 (3)	33 (52)	10 (39)
	Postgraduate	12 (3.4)	9 (4.7)	0 (0)	3 (5)	0 (0)
**Occupation**						
	Employed	125 (36.3)	60 (31.3)	9 (14.3)	46 (73)	10 (39)
	Private business	40 (11.6)	27 (14.1)	5 (8)	8 (13)	0 (0)
	Student	14 (4.1)	0 (0)	0 (0)	10 (16)	4 (15)
	Unemployed	61 (17.7)	14 (7.3)	35 (56)	1 (2)	11 (42)
	Retired	129 (37.5)	108 (56.3)	17 (27)	2 (3)	2 (8)
**Household average monthly income (SR)**						
	<5000	13 (3.8)	43 (22.4)	39 (62)	17 (27)	12 (46)
	5000-10,000	96 (27.9)	52 (27.1)	16 (25)	22 (35)	6 (23)
	10,000-15,000	67 (19.5)	45 (23.4)	5 (8)	12 (19)	5 (19)
	15,000-20,000	39 (11.3)	31 (16.2)	2 (3)	5 (8)	2 (8)
	>20,000	30 (8.7)	21 (10.9)	1 (2)	7 (11)	1 (4)
**Did you receive diabetes education?**						
	Yes	138 (40.1)	80 (41.7)	12 (19)	37 (59)	9 (35)
	No	206 (59.9)	112 (58.3)	51 (81)	26 (41)	17 (65)
**Do you use the Internet?**						
	Yes	134 (38.9)	60 (31.2)	8 (13)	51 (81)	15 (58)
	No	210 (61.0)	132 (68.8)	55 (87)	12 (19)	11 (42)

**Figure 1:**
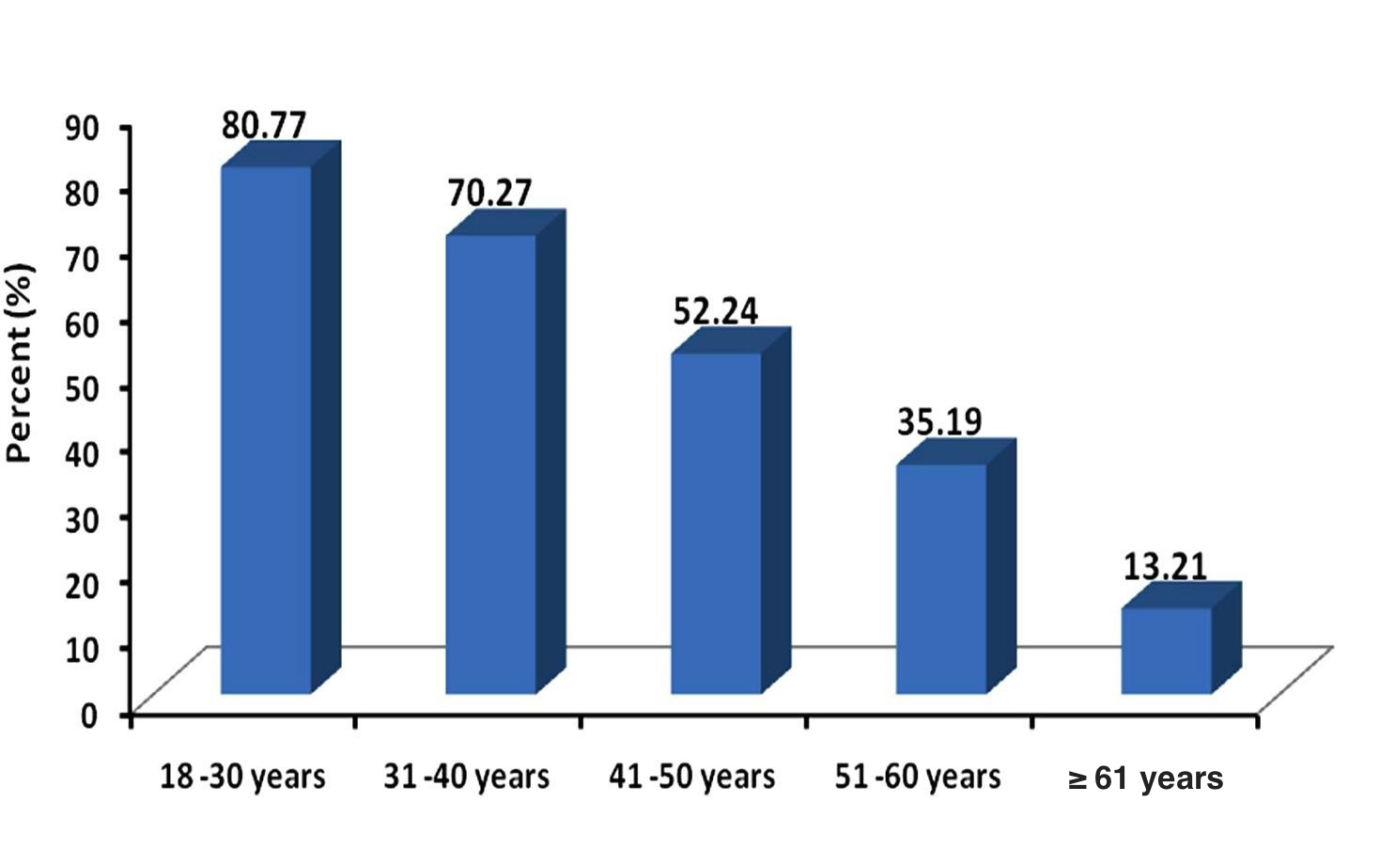
Distribution frequency of Internet use among diabetic patients by age.

**Figure 2:**
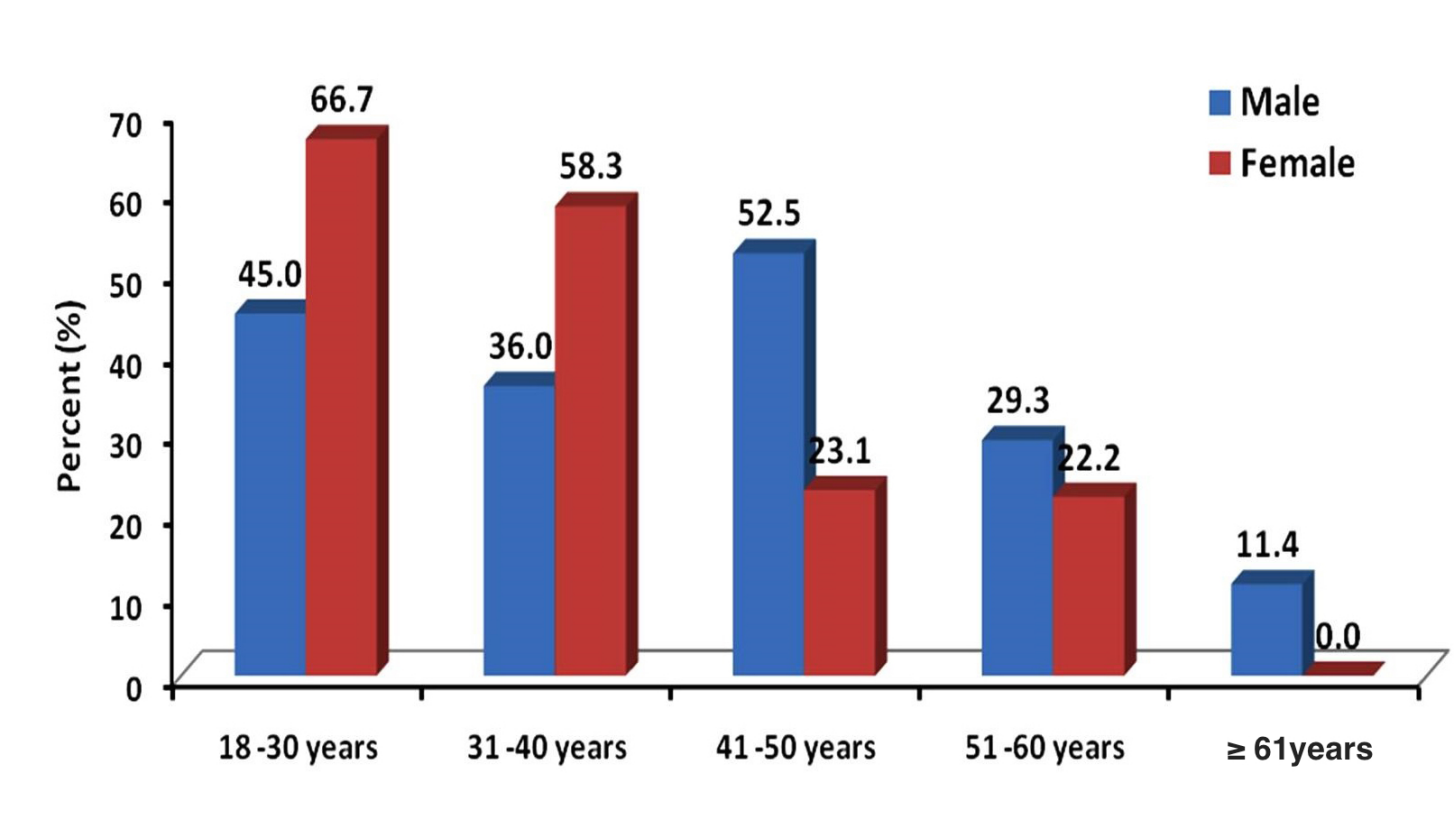
Distribution frequency of health information seekers among diabetic patients by age and gender.

**Figure 3:**
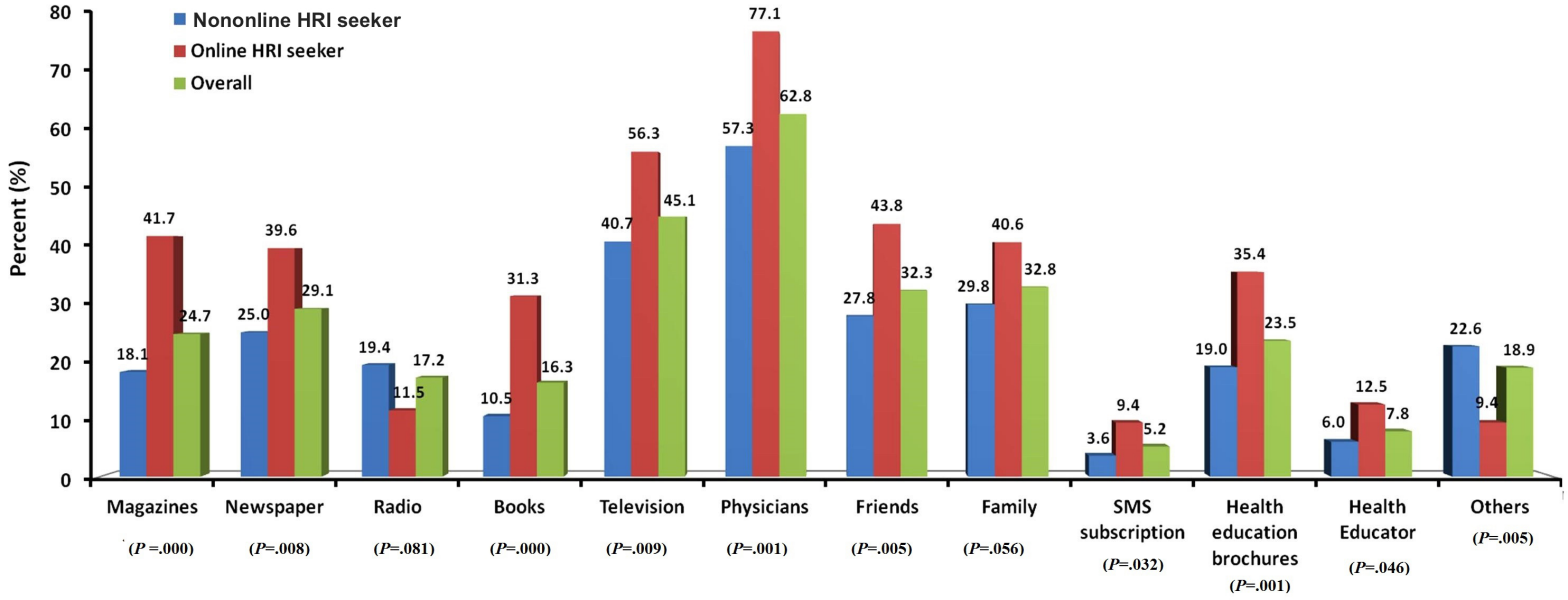
Sources of health-related information for online and nononline health-related information seekers.

**Figure 4:**
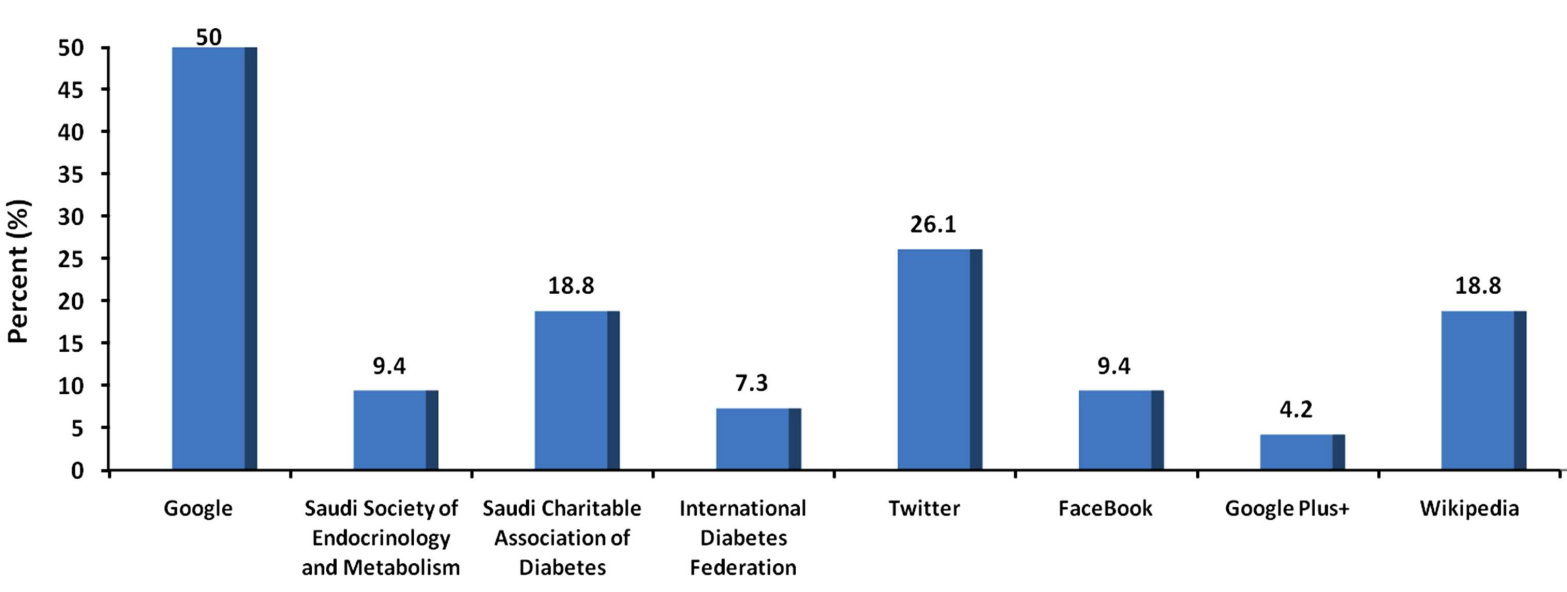
Types and frequency of Internet sites visited by diabetes patients to search for health-related information.

Only 134 of 344 (39.0%) of the interviewed patients were Internet users in general. The majority of Internet users were from younger age groups (Figure 1). Among those who used the Internet, 89.6% (120/134) had access to the Internet at home, 44.8% (60/134) had access at work, and 63.4% (85/120) had access on their mobile phone. Younger patients were more likely to be Internet users than older patients and Internet use declined with increasing age (Figure 1). Similarly, Internet use for health-related information was higher among younger participants. All females (23/23, 100%) who were already using the Internet in general were also using it to seek health-related information, whereas only 65.8% (73/111) of male participants who were already using Internet in general were also using it for health-related information (phi –0.286391, *P*<.001) (Figure 2). But this pattern was lower for the additional age band and no female aged 60 years or older used the Internet for health-related information. Most Internet users (105/134, 78.4%) reported effective (good/very good) skills of Internet searching (Table 2). The majority of online health-related information seekers (76/96, 79%) reported that their main source of information was still their physician. However, only 96 of 134 Internet users (71.6%) reported using the Internet for health-related information with a mean search frequency of 6.4 (SD 9.9) times per month and a median of 2 (IQR 1-5) times per month (Table 2). Among all surveyed participants (N=344), most of the non-Internet users reported that their primary source of health-related information was their physician (216/344, 62.8%) followed by television (155/344, 45.1%), family (113/344, 32.8%), and newspapers (100/344, 29.1%), whereas 66 of 344 (19.2%) stated none for any health-related information sources (Figure 3). It was observed that online health-related information seekers used a variety of health-related information sources and these sources were preferred significantly by online health-related information seekers compared to the seekers of non–health-related information (Figure 4).

**Table 2:** Characteristics of Internet users included in the study.

Characteristics		Total, n (%) n=134	Age band, n (%)	
			≥45 years n=68	<45 years n=66
**I have access to the Internet**				
	At home	120 (89.6)	59 (87)	61 (92)
	At work	60 (44.8)	32 (47)	28 (42)
	On my mobile phone	85 (63.4)	42 (62)	43 (65)
**Frequency of use per month**				
	0-5 times	75 (55.9)	42 (62)	33 (50)
	6-10 times	8 (6.0)	5 (7)	3 (5)
	≥11 times	13 (9.7)	5 (7)	8 (12)
**Rating of searching skills**				
	Very good	51 (38.1)	22 (32)	29 (44)
	Good	54 (40.3)	25 (37)	29 (44)
	Fair	22 (16.4)	15 (22)	7 (11)
	Poor	7 (5.2)	6 (9)	1 (2)
**Have you used the Internet to search for health information?**				
	Yes	96 (71.6)	52 (77)	44 (67)
	Never	38 (28.4)	16 (24)	22 (33)

### General Internet Usage

The majority of online health-related information seekers used Google as the primary search engine to look for the health information (94/96, 98%) followed by Bing (2/96, 2%), whereas no one selected the Yahoo search engine. The criterion for how they selected the link from the search result list was related to the perceived compatibility with the words they searched (62/96, 65%), followed by first link in the search result (25/96, 26%), and lastly URL type (eg, org/gov/edu/com) (12/96, 13%). The most frequent website used by study participants was Google (48/96, 50%) followed by Twitter (25/96, 26%); the Saudi Charitable Association of Diabetes and Wikipedia shared the same percentage (18/96, 19%) (Figure 4). The majority of online health-related information seekers were searching for general health knowledge (64/96, 67%) followed by treatment of health problems (47%, 45/96) (Table 2). Whereas for diabetes-related information, the primary topics the participants were looking for were related to therapeutic diet for diabetes and symptoms of diabetes, followed by diabetes treatment and causes of diabetes (Table 3). Most of the online health-related information seekers (89/96, 93%) reported positive change in their behaviors after seeking online health information.

**Table 3:** Characteristics of online health-related information seekers included in the study.

Characteristics of health-related information seeker		Total, n (%) n=96	Age band, n (%)	
			≥45 years n=52	<45 years n=44
**Where do you start looking for information on the Internet?**				
	MSN	2 (2)	1 (2)	1 (2)
	Google	94 (98)	51 (75)	43 (65)
	Yahoo	0 (0)	0 (0)	0 (0)
	Bing	0 (0)	0 (0)	0 (0)
**What was the primary reason you asked/looked for health information during the past year?**				
	Manage health	45 (47)	20 (29)	25 (38)
	Diagnose health problem	29 (30)	15 (22)	14 (21)
	Info about diseases prevention	26 (27)	17 (25)	9 (14)
	For general health knowledge	64 (67)	36 (53)	28 (42)
	For health and wellness info	33 (34)	25 (37)	8 (12)
	Identify symptoms of health condition	11 (11)	7 (10)	4 (6)
	For answering a specific question	21 (3)	10 (15)	11(17)
	Other (please specify)	3 (3)	2 (3)	1 (2)
**What was the primary type/topic of health information that you asked about or looked for during the past year?**				
	What is diabetes	38 (40)	19 (28)	19 (29)
	Symptoms of diabetes	52 (54)	27 (40)	25 (38)
	Causes of diabetes	48 (50)	23 (34)	25 (38)
	Diagnosis of diabetes	24 (25)	13 (19)	11 (17)
	Side effects of diabetes medications	34 (35)	21 (31)	13 (20)
	Lifestyle management	31 (32)	16 (24)	15 (23)
	Diabetes treatment	50 (52)	30 (44)	20 (30)
	Specific health condition info	9 (9)	9 (13.2)	0 (0)
	Therapeutic diet for diabetes	55 (57)	29 (43)	26 (39)
	Therapeutic diet to lose weight	35 (36)	20 (29)	15 (23)
	Complications of diabetes	31 (32)	19 (28)	12 (18)
	Other (please specify)	2 (2)	2 (3)	0 (0)
**After seeking health information and finding this information, did your health behavior change for the better?**				
	Yes	89 (93)	46 (88)	43 (98)
	No	7 (7)	6 (12)	1 (2)

### Online Health-Related Information Seeking Behavior

Logistic regression was performed to assess the impact of a number of sociodemographic factors on online health-related information seeking behavior (Table 4). The model contained 10 independent variables (sex, age band, marital status, education, income, occupation, diabetes duration, diabetes education, genetic run of diabetes). The full model containing all predictors was statistically significant (*P*<.001) indicating that the model was able to distinguish between respondents who used Internet for health-related information and correctly classified 72.3% cases. The strongest predictor was found to be age band; those using the Internet for health-related information were more than 2.59 times (OR 2.59, 95% CI 0.88-3.15) more likely to be among the lower age group participants. Similarly, marital status and education level were also associated factors for seeking health-related information. Duration of diabetes and familial history of diabetes were negative predictors, suggesting that patients with longer duration of diabetes and a family history of diabetes were less likely to use the Internet for health-related information.

The odds ratio of 0.458 (95% CI 0.119-1.761) for occupation was less than 1, indicating that those who were either retired or unemployed were 55% less likely to use the Internet for health-related information. Even those who reported to have exposure to diabetes education were 4.3% less likely to use the Internet for health-related information compared to nonexposed patients. The mean duration of Internet usage for health-related information seekers and non–health-related information seekers was 7.45 (SD 4.2) times per month and no statistical difference was found comparing health-related information seekers giving mean duration of 7.62 (SD 4.3) times per month and non–health-related information seekers (mean 7.62, SD 4.3 times per month) using Student *t* test on basis of Internet usage. Overall age, gender, marital status, education, income, and diabetes education were found to be important factors associated with online health-related information behavior.

**Table 4:** Logistic regression (N=344 full case data only) modeling odds for nononline health-related information seekers versus online health-related information seekers with sociodemographic details.

Sociodemographic characteristics		Total, n (%) N=344	Not health-related information seekers, n (%) n=248	Health-related information seekers, n (%) n=96	OR (95% CI)	*P*
**Gender**						
	Female	89 (25.9)	66 (26.6)	23 (24)	1.459 (0.737, 2.888)	.29
	Male	255 (74.1)	182 (73.4)	73 (76)	Ref	—
**Age band**						
	≤45 years	89 (25.9)	45 (18.1)	44 (46)	2.593 (0.918, 7.323)	.001
	>45 years	257 (74.7)	203 (81.9)	54 (56)	Ref	—
**Marital status**						
	Never married	26 (7.6)	15 (6.0)	11 (12)	2.036 (0.477, 8.686)	.34
	Married	318 (92.4)	233 (94.0)	85 (89)	Ref	—
**Education**						
	Less than high school	238 (69.2)	194 (78.2)	44 (46)	0.403 (0.219, 0.740)	.52
	University degree	98 (28.5)	54 (21.8)	44 (46)	Ref	—
**Household monthly income (SR)**						
	≤10,000	216 (62.8)	164 (66.1)	52 (54)	0.458 (0.119, 1.761)	.33
	>10,000	127 (36.9)	84 (33.9)	43 (45)	Ref	—
**Occupation**						
	Unemployed	179 (52.0)	127 (51.2)	52 (54)	1.081 (0.325, 3.595)	.90
	Employed	181 (56.6)	121 (48.8)	60 (63)	Ref	—
**Diabetes education**						
	No	202 (58.7)	166 (66.9)	36 (38)	0.406 (0.232, 0.709)	.002
	Yes	122 (35.5)	82 (33.1)	40 (42)	Ref	—
**Duration of diabetes**						
	≤10 years	168 (48.8)	112 (45.1)	56 (58)	0.957 (0.900, 117)	.16
	>10 years	176 (51.2)	136 (54.8)	40 (42)	Ref	—
**Family history of diabetes**						
	No	267 (77.6)	183 (73.8)	84 (88)	0.438 (0.126, 1.528)	.20
	Yes	77 (22.4)	65 (26.2)	12 (13)	Ref	—

### Impact of Health-Related Information Users and Nonusers on Self- Care

Another logistic regression model was performed to assess the impact of seeking online health-related information on self-care among diabetic patients. Table 5 presents the logistic regression analysis or odds of health-related information seekers and nonseekers of self-care health information. The overall model was significantly better in explaining the relationship between online health-related information seekers and self- care. Overall, 4 self-care–related activities were significant factors in the model. Although most of the factors by themselves were not significant factors, they were retained in the model because of their contribution to the overall model as demonstrated by the likelihood ratio test. Removing these factors from the model changed the smaller model significantly from the one that included these factors; therefore, they were retained in the model (Table 5). Out of 12 self-related activities questions, 7 activities showed higher positive association with online health-related information seekers. The strongest association of online health-related information seekers were observed for “their blood glucose check by themselves” and it was found that this check was 4.63 times (OR 4.63, 95% CI 1.86-11.56) more likely to be performed by online health-related information seekers compared to the health-related information nonseekers. With regards to testing for glucose, 28.6% (71/248) of non–health-related information seekers could test it themselves, whereas 93% (89/96) of health-related information seekers could test it themselves (*P*=.001).

For high blood glucose, 68% (65/96) of online health-related information seekers knew what to do correctly, whereas only 44.4% (110/248) of non–health-related information seekers did (*P=*.003). Additionally, online health-related information seekers (33/96, 34%) were more likely to be aware of the importance of exercise and diet on the management of diabetes than non–health-related information seekers (42/248, 16.9%, *P*=.006) (Table 5). There was no statistically significant difference between online health-related information seekers and nonseekers for ophthalmologist and family physician checkups and performing diabetic foot self-exams.

The overall odds ratio (OR 1.56, 95% CI 0.63-3.28) of all self-care questionnaire responses demonstrated that there was no statistically significant difference between those seeking health-related information online and traditional health-related information seekers.

**Table 5:** Logistic regression to predict impact of health-related information seekers and nonseekers on self-health care activities.

Self-care health characteristics			Total, n (%) N=344	Not health-related information seeker, n (%) n=248	Health-related information seeker, n (%) n=96	OR (95% CI)	*P*
**Do you test your blood glucose (sugar) by yourself?**							
	Yes		77 (22.4)	71 (28.6)	89 (92.7)	4.63 (1.86, 11.56)	.001
	No		264 (76.7)	177 (71.4)	7 (7.3)	Ref	—
**How do you treat high blood glucose?**							
	Inject insulin/avoid eating/others		305 (88.7)	219 (88.3)	87 (90.6)	1.21 (0.43, 2.75)	.87
	Don’t know		36 (10.5)	28 (11.3)	9 (9.4)	Ref	—
**Do you wear a medical identification bracelet or necklace?**							
	Yes		11 (3.2)	9 (3.6)	2 (2.1)	1.51 (0.20, 5.60)	.95
	No		330 (95.9)	239 (96.4)	94 (97.9)	Ref	—
**Do you have a glucagon kit at home for severe lows (blood glucose)?**							
	Yes		67 (19.5)	41 (16.5)	27 (28.1)	0.11 (0.63, 1.13)	.12
	No		274 (79.7)	207 (83.5)	69 (71.9)	Ref	—
**In case of severe high blood glucose (sugar)**							
	Do something		175 (50.9)	110 (44.4)	65 (67.7)	0.14 (0.24, 1.32)	.003
	No/don’t know		166 (48.3)	136 (54.8)	31 (32.3)	Ref	—
**What type of treatment that you take to manage diabetes?**							
	**Insulin injections**						
		Yes	157 (45.6)	123 (49.6)	36 (37.5)	2.46 (1.29, 4.69)	.006
		No	184 (53.5)	125 (50.4)	60 (62.5)	Ref	—
	**Diabetes pills**						
		Yes	250 (72.7)	179 (72.2)	72 (75.0)	1.33 (0.67, 2.66)	.41
		No	91 (26.5)	69 (27.8)	24 (25.0)	Ref	—
	**Nonpharmacological treatment (exercises and diet)**						
		Yes	75 (21.8)	42 (16.9)	33 (34.4)	0.94 (0.41, 1.29)	.006
		No	266 (77.3)	206 (83.1)	63 (65.6)	Ref	—
**Do you test for ketones in the urine?**							
	Yes		169 (49.1)	119 (48.0)	52 (54.2)	0.95 (0.56, 1.62)	.85
	No		172 (50.0)	129 (52.0)	44 (45.8)	Ref	—
**Approximately how often do you visit a doctor for your diabetes?**							
	1-4 times a year		328 (95.3)	237 (95.6)	94 (97.9)	1.27 (091, 2.40)	.36
	Never		13 (3.8)	11 (4.4)	2 (2.1)	Ref	—
**How many times do you usually go to ophthalmologist for checking your eyes’ retina?**							
	1-3 visit a year		206 (59.9)	143 (57.7)	65 (67.7)	1.27 (0.38, 1.21)	.19
	Never		135 (39.2)	105 (42.3)	31 (32.3)	Ref	—
**How many times do you usually check your feet by yourself?**							
	Daily/once a week		260 (75.6)	188 (75.8)	75 (78.1)	1.59 (0.80, 3.13)	.18
	Never		81 (23.5)	60 (24.2)	21 (21.9)	Ref	—
**Overall impact**							
	Yes (more health conscious)		173 (50.4)	123 (49.8)	58 (60.5)	1.56 (0.63, 3.28)	.33
	No active health response		168 (48.7)	124 (50.1)	38 (39.5)	Ref	—

##  Discussion

### Principal Findings

This study evaluated the extent of Internet use when searching for health-related information among type 2 diabetes patients visiting inpatient and outpatient clinics at 2 large public University Hospitals in Riyadh, Saudi Arabia. The main finding of this study shows that among diabetic patients, the primary sources of health-related information were physicians followed by television, friends, and magazines. Approximately one-quarter of the sampled diabetes patients were using the Internet for health-related information. The major factors associated with online health-related information seeking behavior were age, gender, marital status, educational level, and exposure to diabetic health education. Overall, these study findings have demonstrated that those seeking online health-related information were more conscious about their diabetes self-care compared to non–health-related information seekers.

In Saudi Arabia, Internet usage has increased rapidly over the past 10 years from less than 3% in 2000 to 60% in 2014; today, the trend shows signs of leveling off, particularly among younger age groups [11,12]. Among the surveyed diabetes patients in this study, only 39.0% (134/344) reported having Internet access and 27.9% (96/344) were online health-related information seekers. The Internet usage among diabetic patients was slightly lower compared to the overall national usage data. Additionally, the percentage of Internet use for health-related information in this study is lower than similar studies performed previously in Saudi Arabia, United States, Switzerland, Italy, and India, although these studies were not performed on patients with specific diseases [2,17-20]. Perhaps because English is not the primary language in Saudi Arabia, knowledge of the English language could be a factor influencing how diabetes patients search for health-related information on the Internet. The current findings also show that majority of the participants search only in Arabic. A greater number of participants were searching in Arabic, which is the native language in Saudi Arabia, whereas only 45% (43/96) of the health-related information seeker participants searched in both Arabic and English. Generally, most of the diabetic patients were elderly [13]. This has been revealed by a high mean age (mean 53.47, SD 13.8 years) in this study’s participants. Generally, relatively older participants are not frequent users of the Internet and other digital devices and, even if they do, they may face some obstacles due to lack of searching skills [21-24].

These study results suggest that the physicians, followed by television, family, newspapers, and the Internet are the primary sources of health-related information. Despite the increasing consumer autonomy with the advent of the Internet, the physician remains one of the most preferred sources of health information in the new media environment, suggesting that more doctors need to explore the Internet as a viable medium for communicating with their patients. These results are concurrent with previous reports [1,2,25].

In a US study conducted in 2007, only 19% of online health-related information seekers searched information at least once a week, whereas one-third of the participants used the Internet at least once a month and individuals with a higher education level searched the Internet for health-related information more than any others [17]. However, in a study from Switzerland, the majority of participants searched for health-related information less than once per month [19]. According to some other studies, age, knowledge of the English language, acuteness or chronicity of the disease, and its severity were associated with the frequency of medical searches on the Internet [18,19]. The use of the Internet to search for health-related information decreased in males with age, whereas the highest rate of Internet use for health-related information among females was younger than age 45 years. Males with higher education and single individuals search for health-related information more than others [18]. Similarly, in this study, Internet searches for health-related information were associated with age, marital status, gender, education, diabetes education, and income. Unmarried individuals, females, those with higher education, and a higher income were found to be the more frequent online searchers for health-related information. These results show how education is an important factor with regards to the use of the Internet in searching for health-related information. This was expected because educated individuals and those who can afford digital devices or computers have greater access to the Internet. The current study suggests that a major factor associated with lower health-related information use among patients with diabetes is their age. Results of the multivariable model used in this study show that this is a continuous effect (OR 2.593, 95% CI 0.918-7.323), with Internet usage decreasing with increasing age. Similar observations were reported in previous studies [2,26]. Thus, improving older adults’ access to and comfort with Internet technologies is central to implementing technology-based solutions to help patients manage their diabetes better.

Online solutions, such as Web portals are increasingly touted as a strategy to improve communication, provide support, and connect to needed services and information for patients with diabetes [21,26,27]. Because the prevalence of diabetes increases with age, particularly type 2 diabetes, the target population with the most to gain from health information technology is older patients. The diabetic patients in this study had a variety of motives and used different websites for their searches. However, the majority of the participants stated that they start their search for health information in Google because they do not know where else to go and the Google generator gives them options to find the links. In addition to Google searches, some patients were also using social sites, such as Twitter, Saudi Charitable Association of Diabetes, Wikipedia, and Facebook, to seek online health information. The use of social media technologies for online health-related information also allows the online social media users to create, distribute, share information, and to consult online rankings and reviews of independent websites [25]. Most of the participants in this study reported that the primary reasons for searching health information were general health knowledge, management of health, and for health and wellness info, respectively. For diabetes-related issues, they were searching for information on diet and symptoms of diabetes.

The current study found that patients searching for health information (89/96, 93%) report positive effects on their self-care behavior to managing their diabetes. Similar findings were reported previously for chronic illness and diabetes patients [15,21,22]. A greater number of online health-related information-seeking participants reported that they were more likely to test their blood glucose (sugar) by themselves as compared to the non-health-related information seekers. Likewise, online health-related information seekers who were testing their blood glucose were more aware about the methods for treating low blood glucose compared to the non-health-related information seekers. Only a fraction of participants who were non-health-related information seekers were aware of how to manage high blood glucose by doing something (eg, take pills, insulin injection, drink water) to alleviate their symptoms. On the other hand, the majority of online health-related information seekers were able to manage themselves for dealing with high blood glucose by doing exercise and following a strict diet, and additionally taking pills, insulin injections, and drinking more water during high blood glucose episodes. The majority of online health-related information seekers were also more likely to be aware of how to manage their disease themselves and to visit an ophthalmologist and a family physician regularly for checkups. The health-related information seekers also checked their feet by themselves on a regular basis. Overall, this study’s findings have demonstrated that those seeking health-related information are more aware and conscious about their health self-care and were able to manage most of the diabetes-related self-care themselves compared to the non-health-related information seekers. Thus, this study’s findings are concurrent with previous reports that explaining the correct uses of the Internet to obtain health-related information can lead to better patient awareness for treatment decisions and increased patient satisfaction, resulting in better medical outcomes and improved self-care behavior [17,27]. Additionally, online health-related information improves the physician-patient relationship and increased patient satisfaction [2]. Similarly, most online health-related information seekers in this study significantly reported that physicians are their primary source of health-related information compared to the nonusers. They usually discuss their disease with their physician to know more about self-care. Interestingly, the majority of the participants were not aware about the quality of the websites and the basic information they provide related to their query. Perhaps much of the information on the Internet could be misleading because anyone can claim medical expertise. Most of the time, information on the Internet is incomplete, out of date, and the public might not be able to select valid information [2,19]. To manage and provide better information to patients, health policy makers should prepare guidelines and strictly keep their eyes on fraudulent and misleading diabetes-related information. To empower patient’s knowledge and self-care, physicians should ask their patients if they are using the Internet to obtain diabetes-related health-related information and provide them with reliable/trusted website information.

### Implications for Practice

This study found that 39.0% (134/344) of participants use the Internet. As a result of this finding and the benefits of using online health-related information for diabetes information, physicians should promote the use of verified and credible diabetes websites, especially those with content in Arabic. Among those who used the Internet, there were only 71.6% (96/134) who searched for health-related information. Physicians may increase this number by promoting the credible and trustworthy websites to the 28.4% (38/134) of participants who use the Internet, but not for health-related information. Also, physicians should educate the more educated patients on how to search correctly and be able to critique health-related information using simple approaches. Because physicians are still the main and most trusted source of health information for most patients (216/344, 62.8%), involving physicians in the process of facilitating the online searching of diabetes information to patients is a strategy worth pursuing. With the positive impact it has on the health of patients, it may be a strategy that should be pursued by physicians.

### Strengths and Limitations

Several studies have examined the general public’s use of the Internet to obtain health-related information, but this study is the first research project to explore online health-related information–seeking behavior among Saudi adult patients diagnosed with type 2 diabetes and its impact on patients’ self-care. This study’s sample size is likely to be representative of the patient population in the primary care clinics and inpatients of one center, but may not be representative of the larger population. In this study, the majority of the participants were male. The main reason for this occurrence could be because the research team consisted of primarily males and females in Saudi Arabia often refuse to be interviewed by them due to cultural and social barriers. However, the response rate was high with only 9.4% (37/344) of patients who were approached declining to participate in the study. Because all participants were from a government hospital, most had relatively low education levels and low monthly income, were retired or unemployed, and the majority were married. The majority of the participants in this study did not receive any diabetic education by attending conferences or campaigns related to diabetes.

### Conclusions

Among diabetes patients, less than half (134/344, 39.0%) of the sampled patients in this study used the Internet for health-related information. The majority of the participants who used the Internet belonged to the younger age group. Among Internet users, female participants were more likely to search for health-related information. The majority of the participants reported that their physician was the primary source of health-related information followed by television. Overall, this study demonstrates that those seeking health-related information are more aware about their health care needs as compared to non–health-related information seekers. These study results suggest that physicians should cooperate with their patients and guide them regarding reliable websites, which provide health information per patient need. To improve the health-related information for patients, health care authorities should publish websites that contain reliable health information in the mother tongue so that patients learn better and are more aware of their health condition. The information must be updated and supervised regularly by health care providers. For those patients who do not use the Internet or cannot read, government organizations responsible for public health issues should make policies to reach them through alternative media, such as television, radio, and newspapers.

## Abbreviations

IDF: International Diabetes Federation
MENA: Middle East and North Africa
